# Retraction: Physiology and Pharmacology of the Cardiovascular Adrenergic System

**DOI:** 10.3389/fphys.2015.00379

**Published:** 2015-11-27

**Authors:** Frontiers in Physiology Editorial Office

The journal has retracted the 4 September 2013 article cited above. Based on information discovered after publication and reported to Frontiers in June 2015, the article was examined, revealing that the complaint was valid and that the article should be retracted, because of an unacceptable level of similarity to another review article by Triposkiadis et al. (2009) published in the Journal of the American College of Cardiology. The retraction of the article was approved by the Field Chief Editor of Frontiers in Physiology. The author does not agree to the retraction or to the notice.
